# The Effects of Using Sodium Alginate Hydrosols Treated with Direct Electric Current as Coatings for Sausages

**DOI:** 10.3390/polym9110602

**Published:** 2017-11-11

**Authors:** Żaneta Król, Dominika Kulig, Krzysztof Marycz, Anna Zimoch-Korzycka, Andrzej Jarmoluk

**Affiliations:** 1 Department of Animal Products Technology and Quality Management, The Faculty of Biotechnology and Food Science, Wroclaw University of Environmental and Life Sciences, Chelmonskiego 37/41, 51-630 Wroclaw, Poland; dominika.kulig@uwpr.edu.pl (D.K.); anna.zimoch-korzycka@upwr.edu.pl (A.Z.-K.); andrzej.jarmoluk@upwr.edu.pl (A.J.); 2 Department of Environment Hygiene and Animal Welfare, The Faculty of Biology and Animal Science, Wroclaw University of Environmental and Life Sciences, Chelmonskiego 38 C, 50-630 Wroclaw, Poland; krzysztof.marycz@uwpr.edu.pl

**Keywords:** sodium alginate, direct electric current, antimicrobial, coating, cytotoxicity, antioxidant, color, sensory evaluation, sausage, quality

## Abstract

We investigated the effect of sodium alginate hydrosols (1%) with 0.2% of NaCl treated with direct electric current (DC) used as a coating on microbial (Total Viable Counts, Psychrotrophic bacteria, yeast and molds, Lactic acid bacteria, *Enterobacteriaceae*), physiochemical (pH, lipid oxidation, antioxidant activity, weight loss, color) and sensory properties of skinned pork sausages or with artificial casing stored at 4 °C for 28 days. Moreover, the cytotoxicity analysis of sodium alginate hydrogels was performed. The results have shown that application of experimental coatings on the sausage surface resulted in reducing all tested groups of microorganisms compared to control after a 4-week storage. The cytotoxicity analysis revealed that proliferation of RAW 264.7 and L929 is not inhibited by the samples treated with 200 mA. Ferric reducing antioxidant power (FRAP) and free radical scavenging activity (DPPH) analyses showed that there are no significant differences in antioxidant properties between control samples and those covered with sodium alginate. After 28 days of storage, the highest value of thiobarbituric acid-reactive substances (TBARS) was noticed for variants treated with 400 mA (1.07 mg malondialdehyde/kg), while it was only slightly lower for the control sample (0.95 mg MDA/kg). The obtained results suggest that sodium alginate treated with DC may be used as a coating for food preservation because of its antimicrobial activity and lack of undesirable impact on the quality factors of sausages.

## 1. Introduction

Methods of active packaging have been developing rapidly [1]. These technologies have been applied on a variety of food products such as fresh fruit, vegetables and meat products because of their potential to ensure food quality and safety. Moreover, protective coatings have been used in the meat industry to prevent microbial contamination, off-flavor due to oxidation, color change and shrinkage [2]. Meat and meat products contain water, protein and essential nutrients with favorable pH to support microbial growth. The broad spectrum of bacteria, yeast, molds and viruses are presented on meat in a range depending on the type of product. The most common spoilage bacteria in meat are *Pseudomonas*, *Acinetobacter*, *Brochotrix thermosphacta*, *Moraxella*, *Enterobacter*, *Lactobacillus*, *Leuconostoc* and *Proteus*. This microorganisms cause degradation of proteins and lipids of meat, which leads to changes in appearance, texture and flavor of products [3]. Spoilage bacteria are harmless to people; however, their consumption in high concentration can cause gastrointestinal disturbances [4]. Meat and meat products may also be contaminated with pathogenic microorganisms, such as *Listeria monocytogenes*, *Salmonella typhimurium*, *Salmonella enteritidis, Escherichia coli* 0157:H7 and *Yersinia enterolitica*, which are responsible for food-borne illnesses and deaths [5]. Vacuum packaging or modified atmosphere packaging (MAP) are methods commonly used to extend the shelf life of products. However, there is an increasing concern about the growth of survival of microaerophilic and/or psychrotrophic pathogens [1]. To inhibit the growth of those microorganisms, chemical substances such as propionic acid, sodium benzoate, benzoic acid, sorbic acid, and potassium sorbate can be used. Nevertheless, due to health concerns related to chemical preservatives, the search for natural biopreservatives in edible film-forming preservation has spurred [6]. For example, chitosan [7], lysozyme [8], nisin [9], bacteriocins [10], rosemary, essential oils and cinnamon oil [11] have been tested. Moreover, the new packaging materials with antibacterial properties, such as chitosan coating treated with cold plasma [12] or gelatin and carrageenan hydrosols incorporated with acid electrolyzed water, have been investigated [13]. In our previous studies we revealed that sodium alginate hydrosols and hydrogels treated with direct electric current have antibacterial properties against pathogenic microorganisms. Additionally, the FT-IR and SEM analysis showed that DC application did not cause undesirable changes in the polymers layer. Moreover, the flow and gelation temperature of hydrosols were not affected by DC. The Texture Profile Analysis (TPA), Swelling Ratio (SR) and color and pH measurement were performed on hydrogels. The results revealed that hydrogels’ properties depend on the type and concentration of polymers and the type of gel. Three types of gels were investigated: control, gels on the basis of hydrosols that were treated with DC and gels treated with DC. The research has shown that using DC the parameters of hydrogels can be changed, which extends their applicability [14,15,16].

Sodium alginate or sodium alginate with food additives such as proteins, pectins or cellulose derivatives is most commonly used to prepare coatings [17]. Alginates are composed of linear chains of 1–4 linked α-l-guluronic (G) and β-d-mannuronic (M) acid residues with free hydroxyl (OH^−^) and carboxylate (–COO^−^) groups located along the backbone [18]. Alginates have the ability to form thermo-irreversible gels. Gelation takes place when exposed to cationic metal ions in solution. Broad availability, biodegradability and low price compared to natural casings make alginates a valued film or coating component [17,19].

The aim of the study was to evaluate how coatings prepared on the basis of sodium alginate hydrosols treated with DC influenced quality of skinned sausages or those with artificial casings during 0, 7, 14 and 28 days of storage.

## 2. Materials and Methods

### 2.1. Material

Alginate FD 125 produced from brown seaward (*Laminaria digitate)* with molecular weight of 140 kDa, particle size max. 2% > 620 μm, mannuronic:guluronic ratio = 1.2 (Dupont GRINSTED^®^, Grindsted, Denmark) was used. Sodium alginate was dissolved in 0.2% (*w/v*) sodium chloride solution by stirring (RW 20 digital, IKA, Staufen, Germany) at 300 rpm for thirty minutes at room temperature. The final concentration of sodium alginate hydrosols was 1% (*w/v*). 

### 2.2. Electrical Treatment of Sodium Alginate Hydrosols

**Figure 1:**
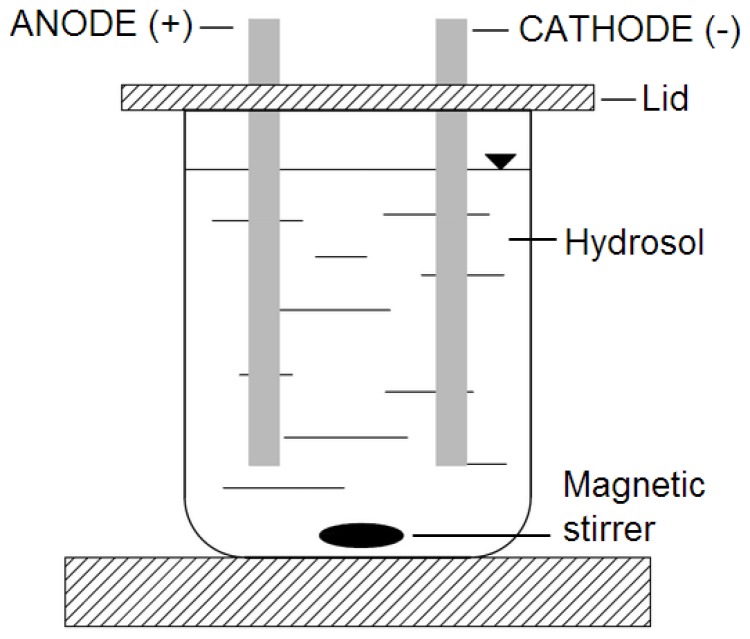
Schematic of the experimental set-up.

Figure 1 shows the experimental set-up used to treat the samples with DC. DC power supply, Major Science MP-SAP (Major Science, Saratoga, NY, USA) was used to provide the current. During all the experiments, the sodium alginate hydrosols were treated with DC of 0 200 and 400 mA for five minutes. During DC application hydrosols were stirred (ECM 5, CAT, Ballrechten-Dottingen, Germany) at 30 rpm. After DC application, polymer solutions were homogenized by homogenizer IKA (T18 basic, Ultra Turrax, Staufen, Germany) for 15 s.

### 2.3. Surface Treatment of Commercial Sausages with the Experimental Material

Pork sausages, vacuum packed, were obtained from the chill cabinet of a local retailer and treated with sodium alginate hydrosol on the day of purchase. The label lists the following ingredients in descending order: pork, water, modified starch, soy protein, spices, extracts of spices, sodium triphosphate, disodium diphosphate, glucose, monosodium glutamate, pork collagen protein, carrageenan, ascorbic acid, sodium nitrite. The sausages were supplied in artificial casing and were up to 14 days from the Use-by Date (UBD) on the day of treatment with sodium alginate. To obtain skinned sausages (S) the artificial casing (AC) was removed. 

The sausages with artificial casing and skinned sausages were dipped for 30 s in sodium alginate hydrosol treated with DC (Table 1) and, after that, were kept over a grid to remove the excess of hydrosol. The sausages (AC/S) covered with sodium alginate were dipped in 0.5M CaCl_2_ solution for 30 s and after transition of sodium alginate hydrosol to gel air-dried in a chamber at 21 °C for 10 min. The covered sausages were then vacuum packed and stored at 4 °C for 28 days. Control sausages were not covered with sodium alginate solution.

### 2.4. Pork Sausage Quality Characterization

All measurements were carried out on the 0, 7, 14, 21 and 28 day of storage.

#### 2.4.1. pH Measurement

Prior to pH measurement, coated/non-coated sausages were homogenized with distilled water (2:8 ratio). Subsequently, Seven Multi™ (model S40, Mettler Toledo, Warsaw, Poland) pH meter equipped with a pH electrode (Inlab Routine Pro, Mettler Toledo) was used twice for pH determination in each sample. 

#### 2.4.2. Microbiological Analysis

Sausage control samples and samples coated with sodium alginate were prepared, packed and stored as described in Section 2.3. The microbiological analysis was conducted according to Kulig et al. [20]. In order to characterize the antimicrobial potential of research material total number of variable counts, psychrotrophs, number of lactic acid bacteria (LAB), yeast and molds and *Enterobacteriaceae* were determined. In accordance to ISO 18593:2004 [21] cotton swabs were used to sampling the surface of coated/non-coated sausages. The 20 square centimeter template was established for swabbing sausages surface. Subsequently, dilutions were prepared as described in ISO 17604:2003 [22]. Colony count technique was used to determine total variable counts [23].

The number of psychrotrophs was determined following method described in ISO 17410:2001 [24]. Yeast and molds culture medium was prepared based on ISO 21527–1:2008 methodology [25]. The number of lactic acid bacteria (LAB) was verified with accordance to ISO 13721:1995 [26]. Enumeration of *Enterobacteriaceae* was carried out according to ISO 5552:2005 [27]. The CFU per milliliter of each sample was calculated as shown in the following equation:

(1) $$N=\frac{\sum C}{\left\{\left(n_{1}\times 1\right)\;+\;\left(n_{2}\times 0.1\right)\right\}\;d}$$

where, *N* is the number of colonies per milliliter of the product (CFU/mL), Σ C is the counted sum of all colonies in all plates, *n*_1_ is the counted number of plates in lower dilution, *n*_2_ is the counted number of plates in higher dilution, *d* is the dilution level corresponding to the first count (*n*_1_). 

The results were presented as log CFU per cm^2^ of sausage surface and calculated using the following equation:

(2) $$N_{s}=\frac{N\times F}{A}$$

where, *N* is the number of colonies per milliliter of the product (CFU/mL), F is the amount of (mL) of dilution fluid, A is the investigated surface (cm^2^).

#### 2.4.3. In Vitro Assessment of Sodium Alginate Hydrogels Treated with DC on Cytotoxicity to Mouse RAW 264.7 Macrophages and L929 Fibroblastic Cell Lines

The test was performed according to the previous protocol [16,28,29]. Sodium alginate was dissolved in sodium chloride solution in the concentration shown in Table 2 by stirring (RW 20 digital, IKA, Staufen, Germany) at 300 rpm for 30 min at room temperature. After DC application, polymer solutions were homogenized by homogenizer IKA (T18 basic, Ultra Turrax, Staufen, Germany) for 15 s. The final concentration of sodium alginate hydrosols was 1% (*w/v*). One milliliter of experimental hydrosols was poured into 24-well plates and inundated with 0.5 ml of 0.5M CaCl_2_. After transition from sol to gel, the excess solution was collected. 

##### Cell Culture

Mouse macrophage RAW 264.7 cell lines and mouse fibroblastic L929 cell lines were purchased from American Type Culture Collection (ATCC, Manassas, VA, USA). RAW 264.7 macrophages were cultured in Dulbecco’s modified eagle medium (DMEM) 4.5 g l-d-glucose, 300 mg/L l-glutamine, and 110 mg/L sodium pyruvate with 10% fetal bovine serum, while L929 cell lines were cultured in Eagle’s Minimum Essential Medium with 10% horse serum. 

For cell incubation temperature of 37 °C and humidified atmosphere of 5% CO_2_ were used. They were observed daily under inverted light microscope (AxioObserverA1, Zeiss, Jena, Germany), images were taken using a Cannon Power Shot, Canon, Tokyo, Japan digital camera.

##### Cell Proliferation Assay

The macrophage cells and fibroblastic cells were inoculated in a 1 mL volume of culture medium and seeded on the tested hydrogels at initial concentration 2 × 10^4^ per well. After 24 and 48 h incubation, the effect of biomaterials was determined. Resazurin based assay (TOX-8, Sigma Aldrich) was used prior to the evaluation of cell proliferation factor (PF). Cell culture media were replaced by 10% resazurin-based dye medium and incubated for two hours. Afterwards, the absorbance of supernatants was spectrophotometrically measured (SPECTRO StarNano, BMG Labtec, Ortenberg, Germany) at 600 nm wavelength, with a distraction of 690 nm of background absorbance. To evaluate the proliferation rate and the number of live cells, a standard curve of a range of cells was calculated with absorbance directly proportional to the number of cells.

##### Cell Viability

Cell viability was observed under light inverted and fluorescent microscope. The cells staining process was preceded by a 30 min immersion in 4% ice cold paraformaldehyde. Afterwards, between every step of preparation, cells were washed with Hank’s balanced salt solution (HBSS). Next, cells membranes were permeabilized for 15 min at room temperature with 0.1% Triton X-100. Subsequently, RAW 264.7 cells were incubated for 30 min in the dark with diamidino-2-phenylindole (DAPI) for 5 min (5 μg/mL) for the analysis of nuclei distribution. Actin filaments of L929 cells were stained in dark conditions using one part of atto-488-labeled phalloidin (Sigma Aldrich, St. Louis, MO, USA) and eight hundreds parts of HBSS solution. After 40 min at room temperature the cells’ nuclei were counterstained with DAPI (1:1000, Sigma Aldrich). Double washing was performed and then cells were observed under inverted phase contrast epifluorescent microscope (Zeiss, Axio Observer A.1, Oberkochen, Germany).

#### 2.4.4. Determination of Thiobarbituric Acid-Reactive Substances (TBARS)

Twenty-five mL of 10% (*w*/*v*) trichloroacetic acid was mixed and homogenized for 30 s using blender with 10 g of grounded pork. After that 5 mL of filtrate was mixed with 2 mL of 20 mM thiobarbituric acid and boiled for 20 min. Mixed solution was centrifuged at 5500 r·min^−1^ for 15 min. The spectrophotometer UV 1800 (Rayleigh Instruments Limited, South Woodham Ferrers, UK) was used to measure absorbance at 532 nm. The obtained results were presented as mg malondialdehyde (MDA) per kg of sample.

#### 2.4.5. Total Antioxidant Capacity (TAC) Measurements

The QUENCHER procedure was used [30]. Ten (±1.0) milligrams of powdered sausage sample were mixed with 10 mL of DPPH or FRAP working solution. DPPH and FRAP working solutions were prepared as described below. Solutions with powdered sausages samples were shaken at 300–400 r·min^−1^. Next, they were stored at room temperature in the dark for 60 and 120 min, FRAP and DPPH assays, respectively. Obtained mixtures were centrifuged at 9200× *g* for 2 min. The 2 mL of supernatant was collected. For DPPH assay the absorbance was measured at 525 nm and for FRAP assay at 593 nm. The results were presented in mmol Trolox Eq./kg of sample.

● DPPH radical solution

Forty mg of DPPH was dissolved in 100 mL of ethanol and diluted with 100 mL of deionized water. To prepare DPPH working solution 800 mL of water/ethanol (50:50, *v*/*v*) mixture was used to dilute 200 mL of stock DPPH solution [30].

● FRAP solution

Twenty five mL 300 mM of acetate buffer were mixed with 2.5 mL 10 mM of TPTZ (2,4,6-tripyridyl-s-triazine) and 2.5 mL 20 mM FeCl_3_ × 6H_2_O to prepare working solution [31].

#### 2.4.6. Weight Loss

The vacuum-packed sausage samples (~20.0 ± 0.01 g) were weight on the 0, 7, 14, 21 and 28 day of storage. Before measurement, sausages were removed from bags. Two parameters were measured:

- Storage losses of weight of sausages (∆*W*_s_), which was calculated using the following equation:
  (3) $$\Delta W_{s}=W_{0}-W_{\mathrm{st}}$$
  where *W*_0_ is the weight of a sausage on the first day of storage and *W*_st_ is the weight at a certain time of storage.
- Cooking losses of weight of sausages (∆*W*_cs_), which was calculated using the equation of:
  (4) $$\Delta W_{\mathrm{cs}}=W_{\mathrm{uc}}-W_{c}$$
  where *W*_uc_ is the weight of uncooked sausage and *W*_c_ is the weight of sausage after cooking for 3 min.

#### 2.4.7. Color Measurement

The reflective colorimeter MINOLTA CR-400 and CR-A33d Light Projection Tube (Ø 22 mm disc, Konica Minolta, Osaka, Japan) set at C illuminant and 2° standard observer was used to measure the color of the sausage samples. The ceramic plate was used for chromameter calibration before each series of measurement (White Calibration Plate CR-A33a, Konica Minolta, Osaka, Japan) to the following coefficients values: Y = 93.8, x = 0.3158, y = 0.3323. The CIELAB values: L* (lightness), a* (+a, red; -a, green) and b* (+b, yellow; -b, blue) were determined. Three locations on each sample were measured. Color differences parameter (ΔE*ab) was calculated from Δ*L*, Δ*a*, Δ*b* parameters (ΔL*a*b = L*a*b at certain time − L*a*b at 0 storage time) values, as follows:

(5) $$\Delta E^{*}ab=\sqrt{{\left(\Delta L\right)}^{2}+{\left(\Delta a\right)}^{2}+{\left(\Delta b\right)}^{2}}$$

#### 2.4.8. Sensory Evaluations

Organoleptic analysis of coated/non-coated sausages was performed at 0, 7, 14, 21 and 28 day of refrigerated storage (4 °C). Nine-point hedonic scale was used in the sensory evaluations of mentioned samples. Sausage samples quality attributes (texture, color, odor and overall acceptability) were evaluated by 20 trained panelists. Sensory evaluation was carried out in a sensory lab under controlled conditions of lighting, temperature and humidity. 

### 2.5. Statistical Analysis

The effects of the current, sodium chloride concentration, storage time, and type of sausage (with artificial casing or skinned) were evaluated. Each experiment was performed in triplicate. Statistica 10 (StatSoft, Cracow, Poland) was used to perform univariate and multivariate analysis of variance (ANOVA). Significant differences between means were assessed by Duncan test with a confidence interval of 95%.

## 3. Results

### 3.1. pH Measurement

**Figure 2:**
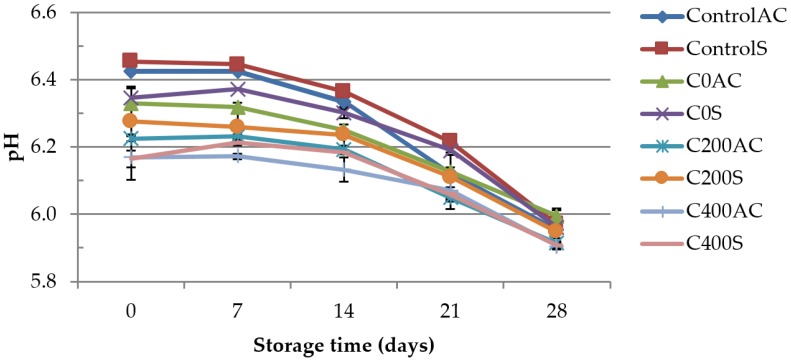
The effect of using experimental sodium alginate casing on the pH of sausages with artificial casing (AC) and skinned sausages (S).

The results have shown (Figure 2) that the experimental sodium alginate coatings affect the pH of sausages significantly. The highest pH on the first day of storage was measured in ControlAC and ControlS samples (6.43 and 6.45, respectively). Similar results for pH values as the control sample have been demonstrated by other investigators in vacuum storage sausages [32,33]. Samples covered with sodium alginate had lower pH. As can be seen, the higher amperage applied in sodium alginate hydrosol, the lower the pH of covered samples. The lowest value of measured parameter was observed for C400AC and C400S variant and was equal to 6.17. These results can be explained by the low pH of hydrogels applied on the sausages. Our previous studies revealed that the pH of sodium alginate hydrogels was lower after applying DC. The pH of 1% sodium alginate hydrogel with 0.2% (*w*/*v*) NaCl treated with DC of 400 mA was about 5.75, while hydrogel not treated with DC had pH ~6.50 [14].The application of DC in hydrosol/hydrogel causes the splitting of water which results in changes in pH [34]. Significant decrease in pH was observed (*p* < 0.05) in all samples throughout storage. The highest values of pH on day 28 of storage was noticed for the C0AC1 variant and was equal to 5.99, while the lowest for the C200 and C400 samples (5.90–5.95). In general, changes in pH vacuum-packed product during storage might result from the production of lactic acid through lactic acid bacteria (LAB) metabolism and carbonic acids formation trough dissolution of CO_2_ into meat aqueous phase [35,36]. However, pH values decreased at a slower rate in samples covered with sodium alginate treated with DC, which can be due to the inhibition of growth of LAB. The use of DC reduced the development of lactic acid bacteria.

### 3.2. Microbiological Analysis

The results relating to the effect of the use of sodium alginate casings on sausage in comparison with the untreated control are shown in Table 3 and Table 4. The total number of bacteria in ControlAC and ControlS samples increased over the course of 28 days of storage from an initial approx. 3.77–3.91 log_10_ CFU/cm^2^ to a maximum of 7.71–7.80 log_10_ CFU/cm^2^. In contrast, the initial values of contamination in samples covered with experimental sodium alginate coatings were lower (2.09–2.78 log_10_ CFU/cm^2^) and reached level between 5.60 and 5.83 log_10_ CFU/cm^2^. According to Mathenjwa the acceptable total microbial quality standard for fresh sausage is 6.00 log_10_ CFU/g [37].

*Enterobacteriaceae* are considered general indicators of the hygiene of food products [20]. The counts remained below detection limit (<1 log CFU/cm^2^) in all samples during 28 days of storage and met the standard of <5.00 log CFU/g for *Enterobacteriaceae* counts [37]. Yeast and molds showed growth up to 1 log cycles during storage period in untreated samples, while in all samples covered with experimental sodium alginate coating the number of survivors was kept below the detection limit during 28 days of storage. These results are in agreement with Garriga et al. [38] and Vercammen et al. [39]. During 30 days of storage (4 °C) of sliced vacuum-packed cooked ham the *Enterobacteriaceae* and yeast and molds counts were not detected. 

Sausages covered with sodium alginate coatings treated with DC showed a significant reduction of at least 2 log cycles for psychrotrophs counts, maintaining the survivors at low levels, around 4 log CFU/cm^2^, after storage. On the last day of storage the number of psychrotrophs count in Control samples were about 6 log CFU/cm^2^. The lowest microbial count of LAB after 28 days of storage was obtained for C400S variant, and was equal to 3.41 log CFU/cm^2^, while the highest for ControlAC and ControlS samples, 7.52 and 7.35, respectively. According to Zimoch-Korzycka et al. [40] and Vermeiren et al. [41] cooked meat products stored in refrigerators under anaerobic conditions are most often spoiled by psychrotrophic lactic acid bacteria (LAB). LAB have tolerance to micro-aerophilic or anaerobic atmospheres. Change of taste (sour, off-flavors, off-odors), color and appearance (milky exudates, greening, slime production) and swelling of the package through gas production may indicate LAB-derived spoilage. Presented research confirmed antimicrobial effect of using sodium alginate treated with DC as a coating for sausages. The type of sausage (with artificial casing AC or skinless S) has no great significance for the microbial stability. 

The antimicrobial activity of electric current has already been described [42,43]. The high-intensity pulsed electric fields cause the irreversible damage of the cell membrane, leading to death of microbial cells [44]. She et al. [45] and Jackman et al. [46] confirmed that also a weak electric current (10, 20, 100 mA) inhibits cell growth in liquid. The research conducted by Król et al. [47] showed that DC of 10–30 mA used on agar plates inoculated with *S. aureus* and *Y. enterocolitica* inhibits the growth of these bacteria. In our previous studies [16] we investigated antibacterial activity of sodium alginate hydrosols treated with DC against *S. aureus*, *L. monocytogenes*, *B. cereus*, *M. luteus*, *E. coli*, *S. enteritidis*, *Y. enterocolitica* and *P. fluorescence*. The results have shown that after applying 400 mA during 5 min in hydrosol with 0.2% NaCl there were no bacteria. The damage of cell wall was presented on the scanning transmission electron microscopy (STEM) micrographs. The bactericidal effect obtained for the sample treated with 200 mA in hydrosol with 0.2% NaCl was similar to the results noticed for variant with 0.1% were 400 mA was applied. These results suggest that the concentration of available chlorine concentration (ACC), which strictly depends on the addition of NaCl, is the most significant antibacterial factor. This statement is supported by other authors [48]. According to Król et al. [16] after applying 200 and 400 mA in the hydrosol layer with 0.2% NaCl, the concentration of ACC was equal to 11.30 and 19.72 mg/L, respectively. Our previous study [15] also revealed that the highest amount of ACC in sodium alginate hydrosol is observed on the 0 day of measurement and it decreased during storage. Strong acid electrolyzed water containing 20–60 mg/L ACC and slightly acidic electrolyzed water with 10–30 mg/L ACC are novel antimicrobial agents that have been used in Japan for several years [34]. Results from Brychcy et al. [13] showed that covering meat with carrageenan and gelatin hydrosols containing acid electrolyzed water (AEW) extend shelf life of meat samples. This author believes that hydrosols incorporated with AEW may be used in food industry as new packaging materials with antimicrobial activity.

### 3.3. In Vitro Assessment of Sodium Alginate Hydrogels Treated with DC on Cytotoxicity to Mouse RAW 264.7 Macrophages and L929 Fibroblastic Cell Lines

**Figure 3:**
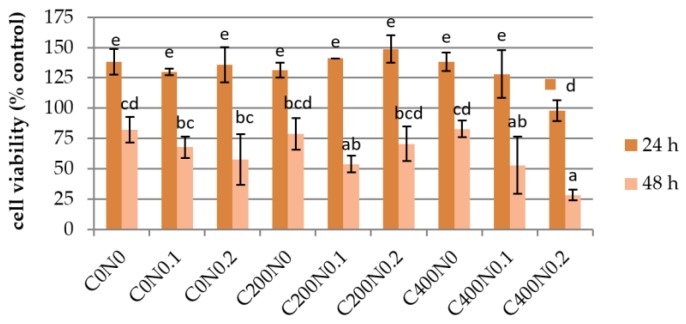
Growth curves showing proliferation rate of RAW 264.7 cells cultured on hydrogel. Vertical bars represent standard deviation. Values followed by different superscript letters (a–e) are significantly different (*p* < 0.05).

**Figure 4:**
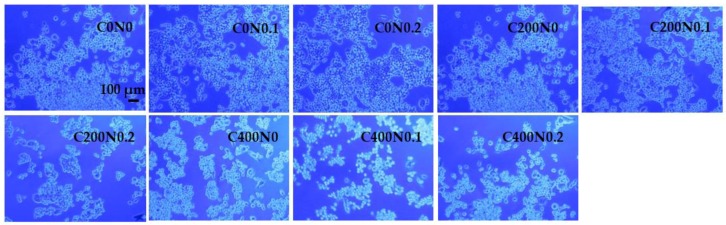
Representative microphotographs of DAPI- staining. RAW264.7 cells were cultured hydrogel materials for 24 h followed by visualization of DNA staining with DAPI using a fluorescence microscope. The data shown are representative of two individual experiments.

**Figure 5:**
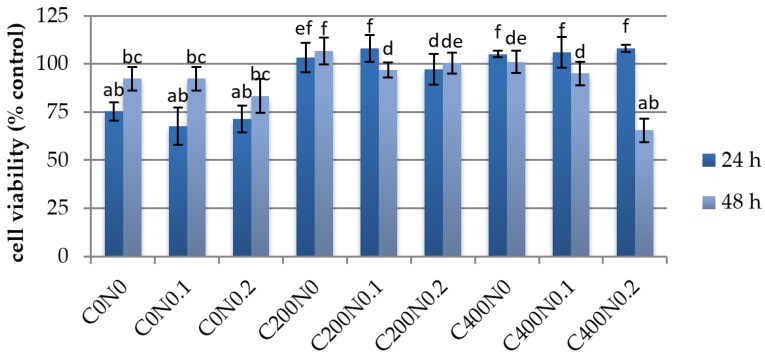
Growth curves showing proliferation rate of L929 cells cultured on hydrogel. Vertical bars represent standard deviation. Values followed by different superscript letters (a–f) are significantly different (*p* < 0.05).

**Figure 6:**
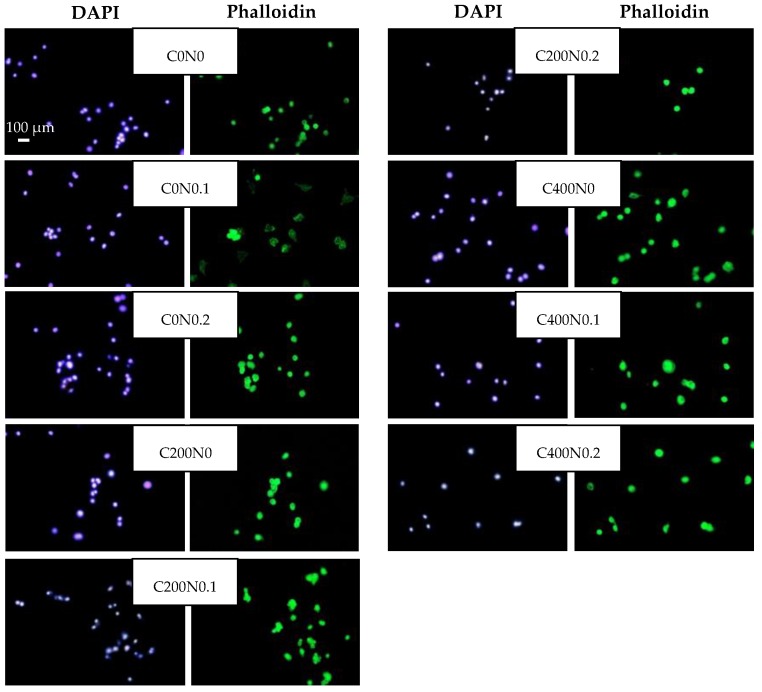
Representative microphotographs of DAPI and phalloidin fluorescent staining. L929 cells were cultured in hydrogel materials for 24 h. The data shown are representative of two individual experiments.

The cytotoxicity effect of sodium alginate hydrogels prepared on the basis of hydrosols treated with DC against mouse RAW 264.7 cells and L929 cells was investigated. The results are presented in Figure 3, Figure 4, Figure 5 and Figure 6. The proper morphology of RAW 264.7 (Figure 4) was observed in all experimental hydrogels. Cells tightly attached themselves and created clusters. In samples treated with 400 mA an irregular form of RAW 264.7 cells can be observed. Some aggregations of nuclei were noticed in all groups. The population of L929 cells is shown in Figure 6. The large flat cells of irregular shape, and small round shaped cells can be seen as well as cells characterized by centrally localized nuclei and well developed cytoskeletons. The cultures on samples treated with DC were evenly spread. After 24 h of cultivation, L929 cells cultured on experimental samples increased in number (Figure 5), whereas in case of samples not treated with DC (C0N0, C0N0.1, C0N0.2) cells slowed down their proliferation. The viability of RAW 264.7 cells cultured on experimental samples was similar, except C400N0.2 variant (Figure 3). After 48 h, RAW 264.7 cells cultured on all samples decreased in number. The lowest cell number was noticed for C400N0.2 variant. According to our previous studies [14] sodium alginate hydrosols with 0.2% NaCl treated with DC had the highest ACC concentration equal to 19.72 ml/L. The ACC is the main cause in antibacterial effects of acid electrolyzed water [49]. The safety of AEW was evaluated previously. Morita’s et al. [50] performed experiments on mice with free access to AEW as drinking water. No undesirable effects were observed. Moreover, Kubota et al. [51] indicated that peritoneal irrigation of experimental perforated peritionis with strong acid water in rats showed no adverse effects. Mokudai et al. [52] and Izumi et al. [53], suggested that it is possible to avoid toxic effect on normal cells with maintained antibacterial activity through the optimization of electric field conditions and addition of sodium chloride. Interestingly, after applying 200 mA and 400 mA in hydrosols with 0 and 0.1% NaCl a greater number of L929 cells (3–23%) were observed in comparison to the samples not treated with DC. It is possible that under specified conditions DC or treated sodium alginate hydrogels can also stimulate the proliferation of L929 cells. The results are in agreement with our previous study [16]. Comparison of the results obtained for antibacterial activity measurements with the cytotoxicity analysis revealed that the C200N0.2 variant results in a satisfactory antibacterial effect without undesirable impacts on normal cells. This statement is also supported by our previous studies [16] were cytotoxicity analyses of sodium alginate hydrosols treated with DC and antibacterial activity against common spoilage bacteria were carried out. On the basis of our research we also have chosen C200N0.2 variant as the most promising. 

### 3.4. Determination of Thiobarbituric Acid-Reactive Substances (TBARS)

**Figure 7:**
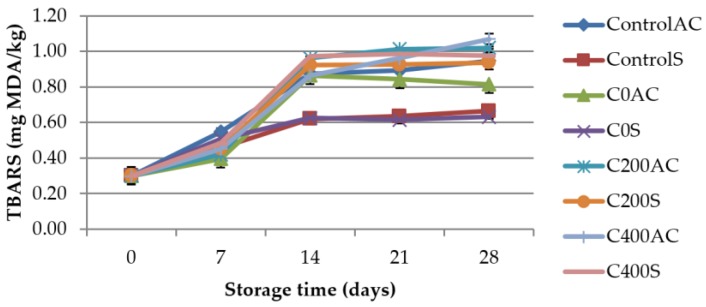
TBARS values for control samples and sausage samples covered with experimental sodium alginate coatings.

We tested the effectiveness of experimental sodium alginate coatings on the oxidative stability of sausage. The results show (Figure 7) that the type of sausage and DC treatment had great importance. On the 0 day of storage the TBARS values of all samples were about 0.30 mg MDA/kg and there were no significant differences between samples. Shimamura et al. [54] confirmed that application of AEW water on meat surface do not changed TBARS values. The increase in TBARS values during storage was observed for all tested samples. The results are in agreement with other authors [55,56]. The lipid oxidation in meat during storage is a natural process although, an activating factor has to be present to initiate the reaction [57]. Lipid oxidation lowers the quality of meat, which in turn becomes unacceptable for consumers. The higher the TBARS values the more advanced lipid oxidation. After 28 days of storage, variant C400AC suffered significant lipid oxidation (1.07 mg MDA/kg). However, it must be emphasized that TBARS value of ControlAC sample was only slightly lower and equal to 0.95 mg MDA/kg. According to Suman et al. [58] TBARS values of 2 mg MDA/kg or grater measured in beef samples are considered to be rancid. The lipid oxidation in food may be initiated by a number of mechanisms [59]. It is possible that the strong oxidizing activity of products generated during electrolysis [42,60,61] lead to slightly faster lipid oxidation. Rahman et al. [62] reported that TBARS values of chicken breasts treated with slightly acid low concentration electrolyzed water (10 ppm of ACC) and strong acid electrolyzed water (50 ppm of ACC) increased during storage but were lower than for the control sample. Shimamura et al. [53] demonstrated that lipid oxidation of meat can be reduced by combination treatment with alkaline electrolyzed water (ALEW) and strong acidic electrolyzed water (StAEW). Our results revealed that the lower the applied amperage, the lower TBARS values. Higher TBARS values of skinned samples covered with sodium alginate treated with DC can be due to direct contact of experimental coatings with sausages. 

### 3.5. Total Antioxidant Capacity (TAC) Measurements

**Figure 8:**
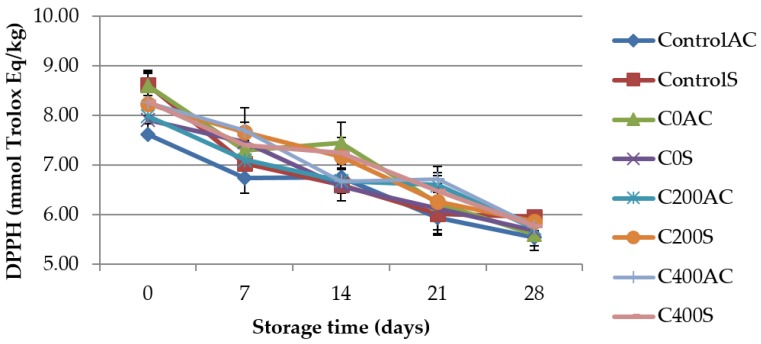
TAC value using DPPH as the antioxidant probe in sausage samples covered with sodium alginate coatings.

**Figure 9:**
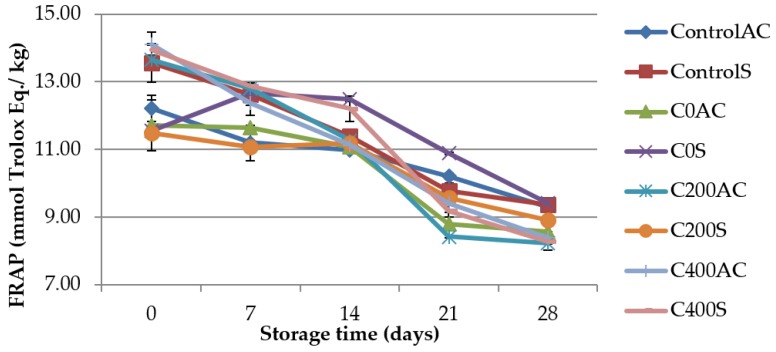
TAC value using FRAP as the antioxidant probe in sausage samples covered with sodium alginate coatings.

QUENCHER procedure [30] was used to measure TAC value of the sausages. The results are shown in Figure 8 and Figure 9. It can be noticed that DPPH and FRAP showed significant difference (*p* < 0.05) with time. DPPH and FRAP values decreased significantly with the storage period which showed that antioxidant activity of sausages decreased during storage. A similar trend of measured parameters has been reported by Arshad et al. [63]. These results can be due to the reduced efficiency of endogenous antioxidants resulting from such factors as increased oxidation in products during storage and thus, could not inhibit free radicals at high percentage [36]. On the 0 day of storage and after a 4-week analysis no significant differences in DPPH value between control and experimental samples were noticed. On the 0 day of storage DPPH values were about 7.61–8.62 mmol Trolox Eq./kg, while after 28 days 5.54–5.97 mmol Trolox Eq./kg. Obtained results are not in agreement with Brychcy et al. [13]. Researchers noticed that after 7 days of storage the DPPH values were higher for meat samples covered with gelatin and carrageenan hydrosols incorporated with AEW than for control samples (without AEW). Different results were obtained in FRAP analysis (Figure 9). Samples covered with sodium alginate treated with DC were characterized by about 9% lower FRAP values than control samples. Our earlier studies [16] showed that DC does not affect the ferric reducing antioxidant power of sodium alginate hydrosols. According to Serpen et al. [30] low values of FRAP may be due to poor ability of meat antioxidants to reduce ferric ion to its ferrous form. Meat is very susceptible to oxidation. To obtain oxidative stability the balance between endogenous pro-oxidant and antioxidant substances is necessary [30]. Factors that promote oxidation include: endogenous pro-oxidant substances (e.g., hemoglobin and other iron porphyrins), polyunsaturated fatty acids (PUFA), cholesterol, protein and pigments, available oxygen. The antioxidant capacity of meat can be due to antioxidant compounds (vitamin E, vitamin C, carotenoids, ubiquinols, polyphenols, cellular thiols), and enzymes (superoxide dismutase, catalase, glutathione peroxidase). Moreover, some proteins and peptides may act as antioxidants. Especially, amino acids and dipeptides such as carnosine and anserine which contribute to the inactivation of free radicals, lipid oxidation catalysts may also counteract the glycation of proteins [64]. Antonini et al. [65] suggest that carnosine (b-alanyl-l-histidine) and anserine (Nb-alanyl-l-methyl-l-histidine) are the principal antioxidants in meat. The oxidative modification of lipids leads to changes in molecules, and generate oxidized lipids-derived aldehydes and peroxides, which are toxic compounds. Therefore, the balance between factors that promote oxidation and those that counteract this process is desirable [66].

### 3.6. Weight Measurement

**Figure 10:**
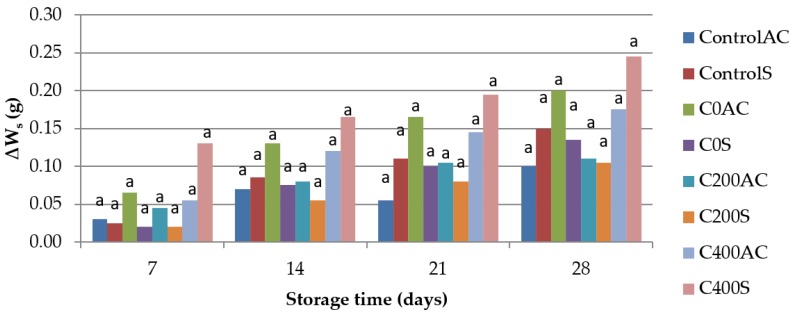
The effect of using sodium alginate coatings on the storage losses of weight of sausage (∆*W*_s_). ^a^ Means with the same letter are not different (*p* < 0.05).

**Figure 11:**
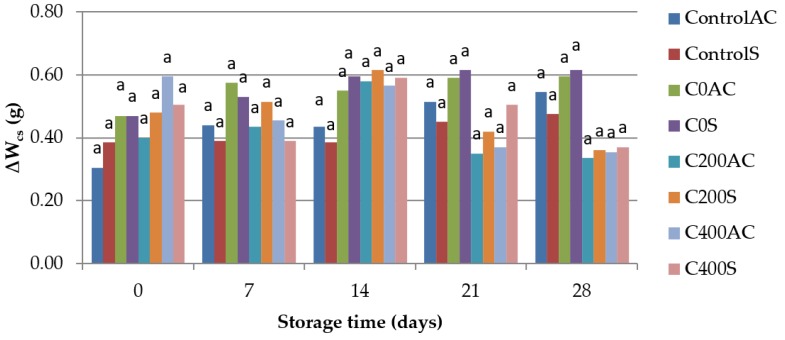
The effect of using sodium alginate coatings on the cooking losses of weight of sausages (∆*W*_cs_). ^a^ Means with the same letter are not different (*p* < 0.05).

Figure 10 shows that during 28 days of storage the sausages did not change their weight. The differences in weight are so small that statistically insignificant (*p* < 0.05). Because of high hydrophilic nature alginates have only a limited barrier to moisture [67]. However, according to Nussinovitch [68] delaying moisture transport by alginate coatings prevent shrinkage, microbial contamination, and change of color of meat and meat products. Cooking loss (∆*W*_cs_) shows meat’s ability to retain water and fat and it is an important indicator during evaluation of profitability of production process [69]. The results obtained in this study are presented in Figure 11. There are no significant differences between cooking losses in the control samples and samples covered with sodium alginate. According to Król et al. [14] sodium alginate hydrogels prepared on the basis of sodium alginate hydrosols treated with DC had lower swelling ability than control samples. Tested hydrogels were less cross-linked in comparison to samples no treated with DC. A less cross-linked film has higher or bigger pores or channel and the diffusion of water-soluble material can be increased [67].

### 3.7. Color Measurement

**Figure 12:**
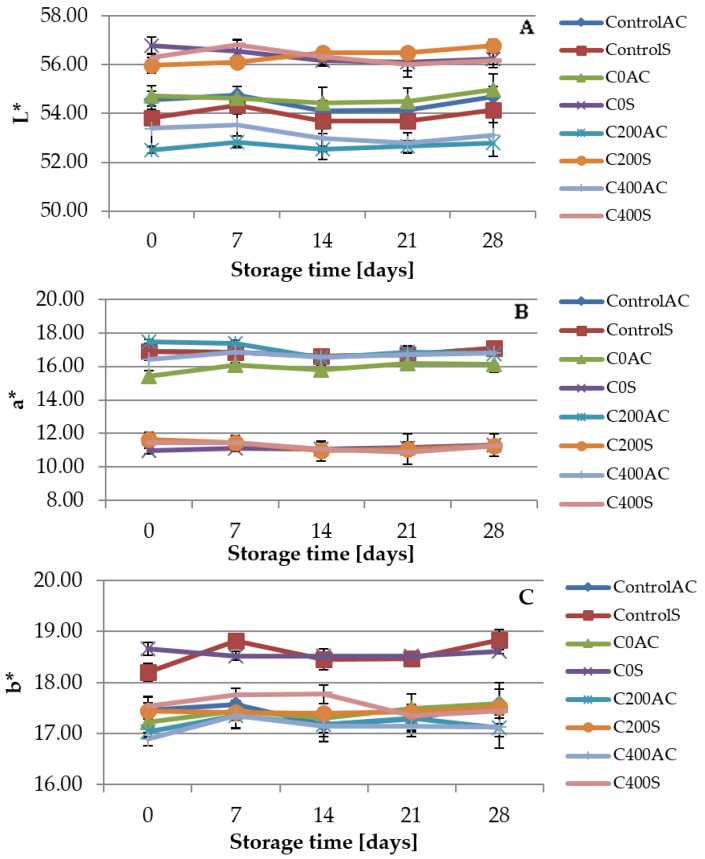
Changes of (**A**) L*; (**B**) a*; (**C**) b* parameters in sausage samples coated with sodium alginate hydrosols.

The results of color measurement are shown in Figure 12. For the AC sausages the differences in measured parameters were not caused by sodium alginate but by the differences in color of artificial casings. Milky color of sodium alginate affected the color parameters of S variants, which is in agreement with Kulig et al. [70]. The lowest value of L* parameter was noticed for ControlS sample, and was equal to 53.81, while the highest for C0S variant (56.77). The same results were obtained for a* parameter. The greatest value of b* parameter was observed for C0S sample (18.66) and the lowest for C200S and C400S variants, 17.44 and 17.53, respectively. After 28 days of storage the lightest were samples covered with sodium alginate (C0S, C200S and C400S), while the reddest was ControlS sample and yellowest were ControlS and C0S0 samples. The same dependency was noticed by Athayde et al. [71]. Authors revealed that the application of different types of electrolyzed water did not affect the red color (a*) of the loins, which indicated that the myoglobin pigment did not undergo major changes, while b* values appeared to be interrelated with the oxidation event. In our work the small changes in color of sausages covered with experimental coatings can be related with TBARS results obtained in the present study. However, the differences were very small and not noticeable by visual inspection. These findings are in agreement with Brychcy et al. [13].

### 3.8. Sensory Evaluations

**Figure 13:**
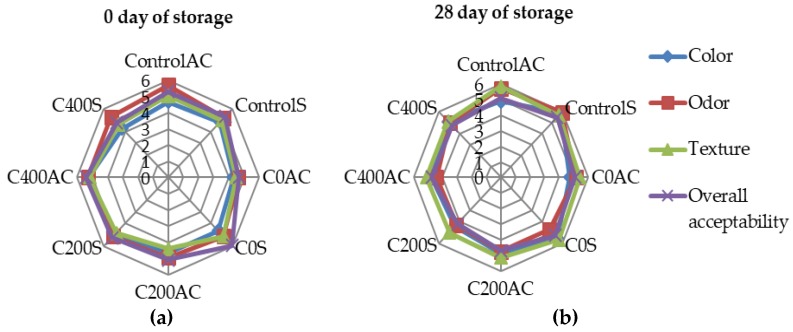
Sensory acceptance of experimental sausages during (**a**) 0 and (**b**) 28 days of storage.

Results of sensory analysis are presented in Figure 13. On the 0 day of storage there were no significant differences in all tested quality factors of all variants. After 28 days of storage sample C200S was assessed lower for color and samples C0AC and C400AC for odor. The highest score for color and odor received ControlS variant. These results can be associated with a less homogeneous structure and the milky color of sodium alginate coatings. Kulig et al. [20] indicated that sensory properties of raw and cooked meat samples coated with Alginate/Chitosan polyelectrolyte complex had no undesirable influence on pork meat products. According to results obtained in color measurement presented in this paper, the differences in color between samples covered with sodium alginate were very small and not noticeable by visual inspection. Moreover, no significant differences in texture and overall appearance of control or experimental samples were noticed. 

## 4. Conclusions

Coatings prepared on the basis of sodium alginate hydrosols treated with direct electric current (DC) are promising materials, which can be used in food industry to prolong lifespan due to their antimicrobial properties and with no undesirable effect on the quality of coated food. Our results revealed that all tested groups of microorganisms were inhibited by about 2 log CFU/cm^2^ on sausage samples covered with experimental sodium alginate coatings. The cytotoxicity analysis showed that toxicity effects of DC could be avoided by using optimized electric field conditions. Moreover, no undesirable impact on quality factors of sausage was observed. After 28 days of storage only slightly lower TBARS values were noticed for control variant than for samples covered with experimental coatings. Radical scavenging (DPPH) and reducing ability (FRAP) assays showed that DC treatment had no influence on antioxidant activity of experimental sausages. There were no significant differences between storage losses and cooking losses in the control samples and samples covered with sodium alginate. Sensory panelists assessed the color of C200S variant and odor of C0AC and C400AC slightly lower, which could be due to the used polymer and not because of DC treatment. Consequently, changes in L* a* b* color parameters were noticed. However, the differences were very small and not noticeable by visual inspection.

## Figures and Tables

**Table 1:** Experimental design.

Run Code Letters	Current (C) (mA)	Type of Sausage (AC/S)
ControlAC *	0	AC
ControlS *		S
C0AC	0	AC
C0S		S
C200AC	200	AC
C200S		S
C400AC	400	AC
C400S		S

AC-sausages with artificial casing; S-skinned sausages; * Control sausages not covered with sodium alginate solution.

**Table 2:** The experimental design used for cytotoxicity analysis.

Run Code Letters	Current (C) (mA)	NaCl (N) (%)
C0N0	0	0.0
C0N0.1		0.1
C0N0.2		0.2
C200N0	200	0.0
C200N0.1		0.1
C200N0.2		0.2
C400N0	400	0.0
C400N0.1		0.1
C400N0.2		0.2

**Table 3:** Populations of microbial groups during refrigerated storage for 28 days of the sausages covered with the experimental sodium alginate coatings.

	Total Viable Counts				
Days of storage	0	7	14	21	28
ControlAC	3.77 ± 0.04 ^c,d^	4.68 ± 0.01 ^d^	5.96 ± 0.00 ^e^	6.97 ± 0.08 ^d^	7.80 ± 0.02 ^b^
ControlS	3.91 ± 0.09 ^d^	4.72 ± 0.01 ^d^	5.76 ± 0.06 ^d^	6.58 ± 0.11 ^c^	7.71 ± 0.06 ^b^
C0AC	3.79 ± 0.01 ^c,d^	4.73 ± 0.06 ^d^	5.76 ± 0.01 ^d^	6.71 ± 0.09 ^c^	7.60 ± 0.20 ^b^
C0S	3.68 ± 0.16 ^c^	4.69 ± 0.01 ^d^	5.74 ± 0.09 ^d^	6.73 ± 0.06 ^c^	7.74 ± 0.11 ^b^
C200AC	2.78 ± 0.02 ^b^	2.92 ± 0.00 ^c^	3.69 ± 0.09 ^c^	4.80 ± 0.04 ^b^	5.83 ± 0.06 ^a^
C200S	2.73 ± 0.05 ^b^	2.78 ± 0.00 ^b^	3.64 ± 0.09 ^b,c^	4.66 ± 0.09 ^a,b^	5.60 ± 0.23 ^a^
C400AC	2.09 ± 0.12 ^a^	2.61 ± 0.11 ^a^	3.49 ± 0.02 ^a,b^	4.56 ± 0.05 ^a^	5.69 ± 0.13 ^a^
C400S	2.13 ± 0.03 ^a^	2.65 ± 0.11 ^a,b^	3.43 ± 0.10 ^a^	4.64 ± 0.04 ^ab^	5.81 ± 0.09 ^a^
	***Enterobacteriaceae***				
Days of storage	0	7	14	21	28
ControlAC	ND ^a^	ND ^a^	ND ^a^	ND ^a^	ND ^a^
ControlS					
C0AC					
C0S					
C200AC					
C200S					
C400AC					
C400S					
	**Yeast and molds**				
Days of storage	0	7	14	21	28
ControlAC	ND ^a^	ND ^a^	ND ^a^	ND ^a^	1.67 ± 0.14 ^b^
ControlS					1.75 ± 0.10 ^b^
C0AC					1.39 ± 0.27 ^b^
C0S					1.78 ± 0.11 ^b^
C200AC					ND ^a^
C200S					
C400AC					
C400S					

Data are expressed in log_10_ CFU/cm^2^; ^a–f^ Values with different letters within the same column differ significantly (*p* < 0.05); ND, no detectable survivors by a direct plating procedure. CFU: colony forming units.

**Table 4:** Populations of microbial groups during refrigerated storage for 28 days of the sausages covered with the experimental sodium alginate coatings.

	**Psychrotrophs**				
Days of storage	0	7	14	21	28
ControlAC	2.83 ± 0.03 ^cd^	3.80 ± 0.14 ^b^	4.75 ± 0.11 ^b^	5.72 ± 0.07 ^c^	6.75 ± 0.01 ^c^
ControlS	2.93 ± 0.05 ^d^	3.66 ± 0.02 ^b^	4.68 ± 0.14 ^b^	5.76 ± 0.10 ^c^	6 65 ± 0.01 ^c^
C0AC	2.82 ± 0.02 ^c^	3.61 ± 0.08 ^b^	4.66 ± 0.04 ^b^	5.69 ± 0.04 ^c^	6.76 ± 0.04 ^c^
C0S	2.75 ± 0.00 ^c^	3.72 ± 0.18 ^b^	4.72 ± 0.10 ^b^	5.71 ± 0.05 ^b^	6.71 ± 0.13 ^c^
C200AC	2.55 ± 0.08 ^b^	2.71 ± 0.11 ^a^	2.90 ± 0.09 ^a^	3.82 ± 0.01 ^b^	4.76 ± 0.10 ^b^
C200S	2.61 ± 0.04 ^b^	2.63 ± 0.23 ^a^	2.87 ± 0.03 ^a^	3.71 ± 0.07 ^a^	4.70 ± 0.04 ^b^
C400AC	2.29 ± 0.04 ^a^	2.47 ± 0.07 ^a^	2.85 ± 0.14 ^a^	3.45 ± 0.08 ^a^	4.64 ± 0.02 ^b^
C400S	2.32 ± 0.05 ^a^	2.50 ± 0.03 ^a^	2.78 ± 0.12 ^a^	3.50 ± 0.15 ^a^	4.38 ± 0.13 ^a^
	**Lactic acid bacteria (LAB)**				
Days of storage	0	7	14	21	28
ControlAC	ND ^a^	1.97 ± 0.04 ^c^	4.83 ± 0.03 ^c^	5.82 ± 0.05 ^d^	7.52 ± 0.06 ^e^
ControlS		1.74 ± 0.04 ^b^	4.93 ± 0.05 ^c^	5.81 ± 0.06 ^d^	7.34 ± 0.12 ^e^
C0AC		1.41 ± 0.13 ^a^	4.75 ± 0.03 ^c^	5.76 ± 0.14 ^d^	6.82 ± 0.06 ^d^
C0S		1.24 ± 0.09 ^a^	4.75 ± 0.02 ^c^	5.75 ± 0.07 ^d^	6.75 ± 0.07 ^d^
C200AC		ND ^a^	1.97 ± 0.00 ^b^	2.77 ± 0.06 ^c^	3.80 ± 0.00 ^c^
C200S			1.74 ± 0.04 ^a^	2.66 ± 0.18 ^ab^	3.66 ± 0.06 ^bc^
C400AC			1.66 ± 0.16 ^a^	2.49 ± 0.03 ^a^	3.50 ± 0.16 ^ab^
C400S			1.59 ± 0.10 ^a^	2.59 ± 0.06 ^a,b^	3.41 ± 0.06 ^a^

Data are expressed in log_10_ CFU/cm^2^; ^a–e^ Means within a column with the same letter are not different (*p* < 0.05); ND, no detected. CFU: colony forming units.
